# Predictive value of an AI model integrating MRI radiomics and clinical features for early recurrence of hepatocellular carcinoma prior to radiofrequency ablation

**DOI:** 10.3389/fradi.2026.1824091

**Published:** 2026-08-18

**Authors:** Beibei Zhang, Wenhua Li, Jing Tang, Ruixin Li, Xiao Wang, Xiaoming Liu, Tianran Li

**Affiliations:** 1 Department of Radiology, the Fourth Medical Center, Chinese PLA General Hospital, Beijing, China; 2 Department of General Surgery, the Fourth Medical Center, Chinese PLA General Hospital, Beijing, China; 3 Beijing United Imaging Intelligent Medical Technology Research Institute, Beijing, China

**Keywords:** artificial intelligence, early recurrence, hepatocellular carcinoma, magnetic resonance imaging, predictive model, radiofrequency ablation, radiomics

## Abstract

**Objectives:**

This study developed an AI-driven radiomics model fusing pre-radiofrequency ablation (RFA) multi-sequence MRI features and clinical variables to predict early recurrence of hepatocellular carcinoma (HCC) after RFA.

**Methods:**

A total of 169 HCC patients who underwent pre-RFA MRI (January 2015–December 2021) were retrospectively enrolled and randomly assigned to a training set (*n* = 135) and an internal hold-out test set (*n* = 34) at an 8:2 ratio using stratified sampling (training: 49 recurrent, 86 non-recurrent; test: 12 recurrent, 22 non-recurrent). Radiomics feature selection involved a three-step strategy of variance threshold filtering, SelectKBest, and least absolute shrinkage and selection operator (LASSO) regression. All data preprocessing, feature selection, and model training were performed exclusively within the training set to prevent information leakage. Support vector machine (SVM), logistic regression (LR), and random forest (RF) classifiers were used to construct radiomics-only, clinical-only, and integrated models. Performance was evaluated using ROC curves (AUC as primary metric), calibration curves, Brier scores, Hosmer–Lemeshow test, and decision curve analysis (DCA). The optimal threshold was determined by the Youden index.

**Results:**

Alpha-fetoprotein (AFP), platelet count (PLT), and tumor location were identified as independent clinical predictors (all VIF < 5). Of 11,320 extracted features, 61.5% (*n* = 6,965) achieved ICC > 0.75, and 16 informative radiomics features were ultimately selected (inter-observer Dice = 0.85 ± 0.06). The radiomics-only models showed robust performance (test AUC: SVM = 0.826, LR = 0.830, RF = 0.826), significantly outperforming clinical-only models (test AUC: SVM = 0.688, LR = 0.706, RF = 0.724; *P* < 0.05). The integrated models achieved further improvement, with the RF-based integrated model demonstrating superior performance (training AUC = 0.975, test AUC = 0.909; *P* < 0.0083 after Bonferroni correction). Calibration showed good agreement (Brier score = 0.143, Hosmer–Lemeshow *P* = 0.367), and DCA demonstrated net clinical benefit across threshold probabilities of 15%–70%. At the optimal threshold (0.42), the RF-based integrated model achieved sensitivity = 83.3%, specificity = 90.9%, PPV = 83.3%, NPV = 90.9%, and accuracy = 88.2%.

**Conclusion:**

Preoperative MRI-based radiomics features combined with clinical variables effectively predict early post-RFA HCC recurrence. The integrated radiomics-clinical model outperforms both clinical-only and radiomics-only models, with the RF classifier yielding optimal performance, providing a reliable tool for individualized treatment decision-making.

## 1 Introduction

Hepatocellular carcinoma (HCC) ranks as the sixth most commonly diagnosed malignant tumor worldwide and the third leading cause of cancer-related mortality, presenting a severe challenge to global public health due to its poor clinical prognosis (1). Surgical resection is the first-line curative treatment for early-stage HCC (2), yet the majority of patients are ineligible for hepatectomy due to underlying liver dysfunction such as cirrhosis, hepatic decompensation, portal hypertension, and abnormal liver volume, which highlights the irreplaceable role of non-surgical interventional therapies in the clinical management of HCC (3, 4). Radiofrequency ablation (RFA) has emerged as a cornerstone treatment for unresectable early and intermediate HCC, owing to its minimal invasiveness, high safety, and favorable short-term efficacy (5). However, the high rate of early post-RFA recurrence remains a major bottleneck restricting the long-term survival of HCC patients, with recurrence manifesting as either local tumor progression or intrahepatic distant metastasis.

Numerous clinical factors have been identified as risk predictors for post-RFA HCC recurrence, including demographic characteristics (age, gender), clinical history (prior HCC treatment), liver disease status (cirrhosis severity, etiology), tumor-related features (location, size, number, differentiation grade, proximity to vital structures), procedural factors (ablation duration, safety margin adequacy), and laboratory biomarkers (alpha-fetoprotein [AFP], protein induced by vitamin K absence or antagonist II [PIVKA-II], liver function indices) (6, 7). Accurate preoperative prediction of early recurrence risk is of great clinical significance for interventional oncologists to conduct individualized prognostic evaluation, formulate rational surveillance protocols, and design targeted adjuvant therapeutic strategies (8).

HCC recurrence is closely associated with the invasive biological behavior of tumor cells, which is influenced by multiple tumor-related factors such as the number of lesions, tumor size, differentiation grade, microvascular invasion (MVI), and Ki-67 proliferation index (9, 10). Tissue biopsy is the gold standard for obtaining tumor pathological information, but it is limited by its invasiveness, sampling bias, and inability to reflect the overall heterogeneity of the tumor. Magnetic resonance imaging (MRI) possesses excellent soft tissue resolution, multi-parametric imaging capabilities, and non-ionizing radiation properties, enabling detailed characterization of tumor morphology, anatomical relationships, and functional features. Functional MRI sequences such as diffusion-weighted imaging (DWI) can reveal the microscopic pathophysiological changes of tumors and have shown promising potential in predicting post-treatment recurrence of HCC (11). The combination of DWI and dynamic contrast-enhanced MRI (DCE-MRI) can further improve the accuracy of evaluating tumor biological behavior and recurrence risk (12).

Conventional visual analysis of MR images only captures subjective and qualitative morphological features of tumors, failing to fully reflect the intrinsic heterogeneity and spatial complexity of HCC. In contrast, radiomics is a novel quantitative imaging technology that enables automated and high-throughput extraction of numerous quantitative features from medical images, which can comprehensively characterize the tissue composition, texture, and morphological characteristics of tumors (13). Combined with artificial intelligence (AI) algorithms, radiomics can realize large-scale high-dimensional data analysis and modeling, providing a new approach for objective diagnosis, prognostic evaluation, and treatment decision-making of malignant tumors (14). Accumulating studies have confirmed the value of MRI-based radiomics in predicting post-RFA recurrence of HCC (15); for example, Zhang et al. developed a radiomics nomogram based on multi-parametric MRI that achieved high accuracy in predicting early recurrence of small HCC after RFA (16). Several clinical nomograms and prognostic scoring systems, such as the Cancer of the Liver Italian Program (CLIP) and the Barcelona Clinic Liver Cancer (BCLC) staging systems, have been used for HCC prognosis assessment; however, these traditional tools lack the quantitative tumor heterogeneity information provided by radiomics and have limited accuracy for individualized recurrence prediction. However, most existing studies focus on post-RFA radiomics features, while preoperative radiomics models for predicting early recurrence remain under-explored. In addition, the interpretability and clinical applicability of radiomics models need to be further improved by integrating clinical variables with high prognostic value.

Therefore, this study aimed to develop an AI-driven integrated predictive model by fusing pre-RFA multi-sequence MRI radiomics features and clinical variables, and to compare the predictive performance of different machine learning classifiers, to provide a reliable quantitative tool for preoperative prediction of early post-RFA recurrence of HCC and to guide clinical individualized treatment.

## 2 Materials and methods

### 2.1 Patient selection and ethical approval

This retrospective cohort study was conducted in accordance with the ethical principles of the Declaration of Helsinki (1964) and its subsequent amendments. The study protocol was approved by the Institutional Ethics Committee of the Fourth Medical Center, Chinese PLA General Hospital (Approval No. 2024KY027-KS001). Written informed consent was obtained from all enrolled patients or their legal guardians prior to any clinical examination and interventional treatment.

**Figure 1:**
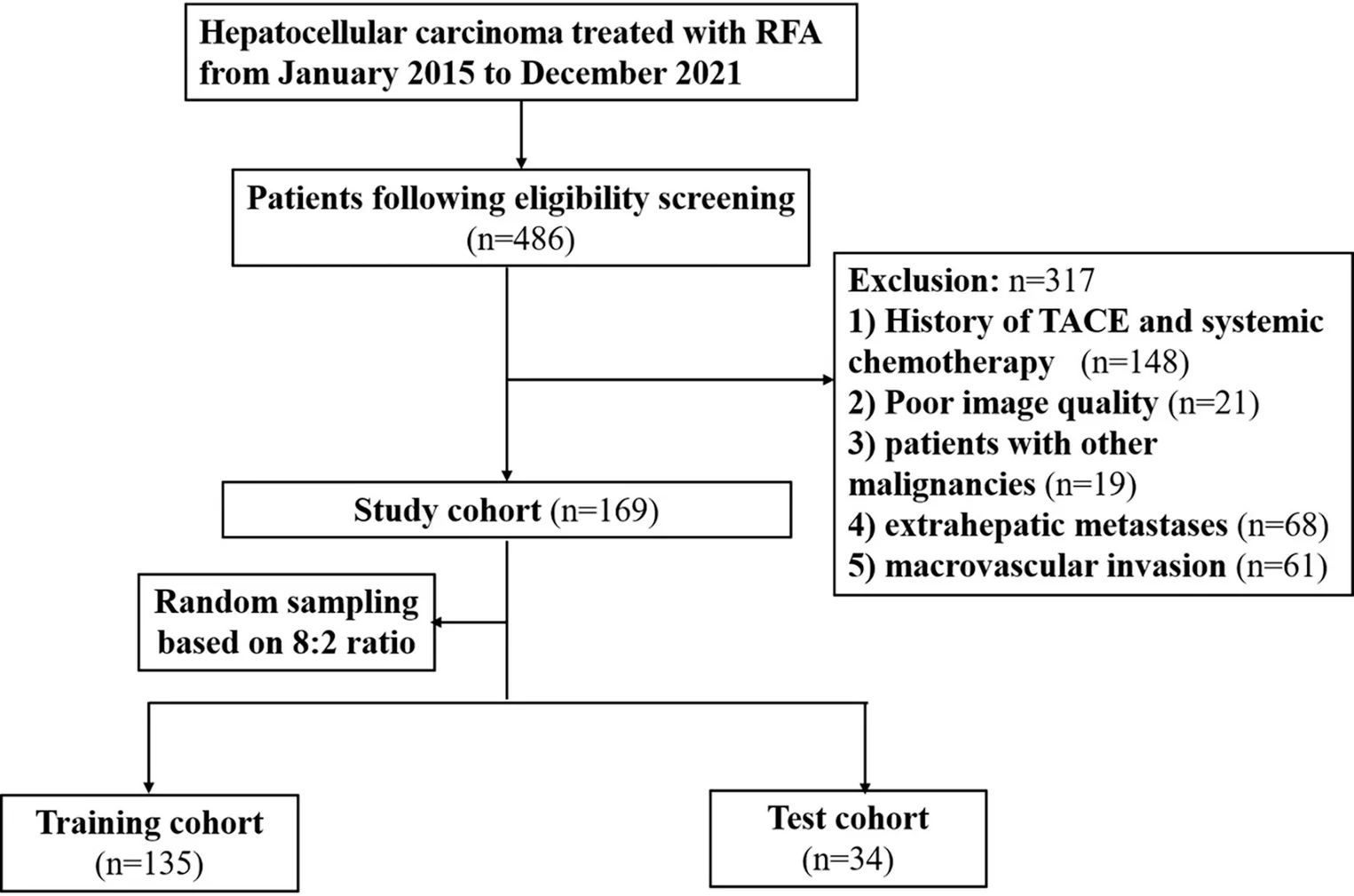
Flow chart of patient enrollment. A total of 486 hepatocellular carcinoma (HCC) patients who underwent radiofrequency ablation (RFA) between January 2015 and December 2021 were initially screened. Patients were excluded for the following reasons: prior transarterial chemoembolization (TACE) or systemic chemotherapy (*n* = 148), macrovascular invasion (*n* = 61), extrahepatic metastases (*n* = 68), other concurrent malignancies (*n* = 19), and significant MRI artifacts affecting diagnostic evaluation (*n* = 21). Finally, 169 eligible patients were enrolled and stratified by recurrence status and randomly assigned to a training set (*n* = 135, 80%) and an internal hold-out test set (*n* = 34, 20%) for model construction and validation.

A retrospective search of the institutional radiology and clinical database was performed to identify HCC patients who were diagnosed and treated at the Fourth Medical Center, Chinese PLA General Hospital from January 2015 to December 2021. The inclusion criteria were as follows: (1) HCC diagnosis confirmed by either imaging findings in accordance with the American Association for the Study of Liver Diseases (AASLD) guidelines (17) or histopathological examination; (2) single tumor with maximum diameter ≤5 cm or multiple tumors with each lesion ≤3 cm; (3) complete pre-RFA multi-sequence MRI data and clinical laboratory examination records available; (4) regular follow-up for at least 1 year after RFA with complete follow-up data. The exclusion criteria were: (1) prior anti-tumor treatment including transarterial chemoembolization (TACE), systemic chemotherapy, or radiotherapy; (2) incomplete postoperative follow-up data or loss to follow-up; (3) presence of macrovascular invasion (e.g., portal vein tumor thrombus) or extrahepatic metastases at baseline; (4) significant MRI artifacts that affected image analysis and feature extraction; (5) combined with other malignant tumors. A total of 169 HCC patients who met all the above criteria were finally enrolled in this study. The flow chart is shown in Figure 1.

The exclusion of 317 of 486 screened patients (65.2%) is justified as follows: 148 patients were excluded for prior TACE or systemic chemotherapy because these patients had more advanced disease requiring combination therapy, and their recurrence patterns differ from those of treatment-naive HCC, which would introduce confounding if included; 61 patients were excluded for macrovascular invasion, 68 for extrahepatic metastases, and 19 for other concurrent malignancies; the 19 patients with other concurrent malignancies were excluded to avoid confounding from competing causes of mortality; 21 patients were excluded for significant MRI artifacts (mainly due to respiratory motion and susceptibility artifacts near the dome of the liver) that precluded reliable radiomics feature extraction. These exclusions were necessary to ensure a homogeneous study population focusing on early-stage HCC treated with RFA as primary therapy.

### 2.2 RFA procedure

All patients underwent laparoscopic or percutaneous RFA under real-time ultrasound guidance using the Cool-tip™ RF Ablation System (Medtronic, MITG, CO, USA). The procedures were performed by a senior hepatobiliary interventional surgeon with more than 20 years of clinical experience in interventional oncology. Ablation was performed in impedance control mode using a 17-G cooled-tip electrode with an exposed tip length of 2 cm, 3 cm, or 4 cm according to the tumor size and location. Internal electrode cooling was achieved through continuous circulation of sterile cold water at a flow rate of 80–140 mL/min, and the total ablation time was set to 12 min for each lesion. If the impedance increased rapidly during the procedure, the power output was automatically suspended and resumed after the impedance decreased to the normal range, with the maximum output power controlled below 10 W. After the ablation was completed, the needle tract was coagulated at 70–90 °C to reduce the risks of postoperative bleeding and tumor seeding along the needle tract.

Safety margin adequacy was assessed on contrast-enhanced MRI performed 1 month after RFA. Ablation margins of ≥5 mm were achieved in 126/169 patients (74.6%), while 43 patients (25.4%) had margins <5 mm. Complete ablation was confirmed on the 1-month follow-up MRI in 161/169 patients (95.3%), and repeat ablation was required in 8/169 patients (4.7%) due to residual viable tumor, and all 8 patients achieved complete ablation after the repeat session. Ablation margin <5 mm was significantly associated with early recurrence (OR = 2.05, 95% CI: 1.01–4.16, *P* = 0.044). However, ablation margin was not included as a variable in the predictive model because it is a post-procedural parameter not available preoperatively, which aligns with the study's goal of preoperative prediction.

### 2.3 Pre-RFA MRI examination

All patients underwent baseline pre-RFA abdominal MRI examinations using a 3.0 T whole-body MRI scanner (Discovery 750, GE Healthcare, USA; or Skyra, Siemens Medical Solutions, Germany) with a phased-array abdominal surface coil. The standardized imaging protocol included T2-weighted imaging (T2WI), DWI (*b*-values = 0 and 800 s/mm^2^), in- and opposed-phase T1-weighted imaging (T1WI), and DCE-MRI. Detailed imaging parameters for each sequence are shown in Table 1. The scanner distribution was as follows: GE Discovery 750 (*n* = 98, 58.0%) and Siemens Skyra (*n* = 71, 42.0%). The distribution was balanced between the training and test sets (*P* = 0.82) and between the recurrence and non-recurrence groups (*P* = 0.65).

**Table 1:** Sequence parameters for upper abdominal 3.0 T MR imaging.

	GE Healthcare				Siemens Skyra			
Parameter	Precontrast T2WI	Precontrast In/out phase	Precontrast DWI	DCE (three phases)	Precontrast T2WI	Precontrast In/out phase	Precontrast DWI	DCE (three phases)
Sequence type	2D Propeller FS	3D SPGR Breath hold	2D SS-EPI Respiratory triggered	3D LAVA-Flex Breath hold	2D FSE FS	3D SPGR Breath hold	2D SS-EPI Respiratory triggered	3D VIBE
No. of sections	32	48	24	60	32	48	24	60
Section thickness (mm)	6.0	5.0	6.0	5.0	6.0	5.0	6.0	5.0
Intersection gap (mm)	0.5	0	0.5	0	0.5	0	0.5	0
Field of view (cm^2^)	38 × 31	38 × 31	38 × 31	36 × 28.8	38 × 31	38 × 31	38 × 31	36 × 28.8
Matrix	320 × 320	320 × 224	160 × 128	320 × 128	320 × 320	320 × 224	160 × 128	320 × 128
TE (ms)	69	Min/2.5	Minimum	Minimum	79	Min/2.5	Minimum	Minimum
TR (ms)	9,474	4.2	…	3.8	4,000	4.1	…	3.8
Bandwidth (kHz)	62.5	166.67	250.0	200.0	62.5	166.67	250.0	200.0
*b* values (s/mm^2^)			0,800				0,800	

DWI, diffusion-weighted imaging; T2WI, T2-weighted imaging; DCE, dynamic contrast-enhanced T1 fat suppressed sequences; TR, repetition time; TE, echo time; Propeller, periodically rotated overlapping parallel lines with enhanced reconstruction; SS-EPI, single-shot echo planar imaging; LAVA, liver acquisition with volume acceleration; FSE, fast spin echo; VIBE, volume spoiled gradient-echo sequence.

For DCE-MRI, a gadolinium-based contrast agent (Magnevist, Bayer Healthcare, Germany) was administered intravenously at a dose of 0.1 mmol/kg body weight with an injection rate of 2.0–2.5 mL/s, followed by a 20 mL saline flush. Dynamic contrast-enhanced images were acquired in three phases: arterial phase (22–25 s after contrast agent injection), portal venous phase (50–70 s), and delayed phase (180–240 s).

### 2.4 MR image analysis and annotation

#### 2.4.1 Conventional MRI feature evaluation

Two senior abdominal radiologists with more than 10 years of experience in hepatobiliary imaging independently performed retrospective visual analysis of pre-RFA MR images, with both readers blinded to the patients' clinical outcomes and each other's evaluation results. All images were reviewed in a random order to avoid selection bias. The conventional MRI features evaluated included: (1) number of lesions (single/multiple); (2) maximum lesion diameter (≤3 cm or >3 cm); (3) tumor location (classified as “special location” if the tumor was adjacent to critical structures including the diaphragm, gallbladder, gastrointestinal tract, intrahepatic bile ducts, porta hepatis, or subcapsular region; otherwise classified as “ordinary location”); (4) presence or absence of tumor capsule; (5) presence or absence of intratumoral fat; (6) presence or absence of underlying liver cirrhosis.

#### 2.4.2 Radiomics image annotation

Regions of interest (ROIs) were manually delineated slice by slice by two trained radiologists using ITK-SNAP software (version 3.8.0, http://www.itksnap.org) on the pre-RFA multi-sequence MR images, including T2WI, DWI, and DCE-MRI (arterial phase, portal venous phase, delayed phase). Volumetric regions of interest (VOIs) were constructed by stacking the slice-by-slice ROIs to cover the entire tumor parenchyma, with strict exclusion of adjacent blood vessels, bile ducts, satellite nodules, and normal liver tissue. All VOI segmentations were independently reviewed and revised by a senior abdominal radiologist with more than 20 years of experience to ensure the accuracy and consistency of tumor segmentation. The mean Dice similarity coefficient between the two observers' VOIs was 0.85 ± 0.06 (range: 0.72–0.94), indicating good spatial overlap. No cases were excluded due to severe inter-observer disagreement. In cases of disagreement, final segmentation decisions were made by the senior radiologist through consensus review with the two primary readers.

### 2.5 Radiomics feature extraction and selection

Radiomics feature extraction and subsequent selection were performed using the United Imaging Intelligent Scientific Research Platform System V1.0 (Beijing United Imaging Healthcare Co., Ltd., China). Prior to feature extraction, all MR images were preprocessed using Z-score normalization to eliminate the influence of different imaging scanners and parameter settings on feature values, and resampled to a unified voxel size using the B-spline interpolation algorithm to ensure radiomics feature consistency. The resampled voxel size was 1.0 × 1.0 × 1.0 mm^3^. Gray-level discretization was performed using a fixed bin width of 25. The filter types included original (no filter), wavelet, and LoG (Laplacian of Gaussian) filters. The radiomics feature definitions and extraction process followed the Image Biomarker Standardisation Initiative (IBSI) guidelines. The platform uses PyRadiomics-compatible algorithms, ensuring reproducibility of feature extraction. A total of 2,264 quantitative radiomics features were extracted from the 3D VOIs of each sequence for each patient, including first-order statistical features, texture features, shape features, and high-order transformation features, with a total of 11,320 radiomics features extracted from the five MRI sequences (T2WI, DWI, arterial phase, portal venous phase, delayed phase).

The intraclass correlation coefficient (ICC) was used to evaluate the inter-observer consistency of radiomics features extracted by the two radiologists. Features with ICC > 0.75 were considered to have good inter-observer consistency and were retained for subsequent analysis. Of the 11,320 initially extracted features, 61.5% (*n* = 6,965) achieved ICC > 0.75 and were retained. A three-step hierarchical feature selection strategy was adopted to reduce the dimensionality of high-dimensional radiomics features and screen out the most informative features for model construction: (1) Variance threshold filtering: features with extremely low variance (close to 0) were eliminated to remove non-informative features; (2) SelectKBest algorithm: features with *P* < 0.05 were retained based on the *F*-test to screen out features significantly associated with early HCC recurrence; (3) LASSO regression: LASSO regression with L1 regularization was used for further feature selection, which can shrink the coefficients of irrelevant features to 0, effectively reducing feature dimensionality and avoiding overfitting, which is particularly suitable for the analysis of high-dimensional radiomics data (18). The optimal regularization parameter *λ* was determined using 10-fold cross-validation, and the final set of radiomics features with non-zero coefficients was selected as the optimal radiomics signature for model construction.

The hierarchical 3-step strategy was designed to progressively reduce dimensionality while preserving informative features: variance threshold filtering first removes non-informative features; SelectKBest with *F*-test then identifies features significantly associated with the outcome; LASSO with L1 regularization handles multicollinearity and selects the most parsimonious feature set. This approach was chosen over elastic net because the primary goal was feature selection rather than coefficient estimation, and LASSO's inherent sparsity is advantageous for clinical interpretability. Feature stability was assessed using bootstrap resampling (1,000 iterations), with 14 of the 16 final features selected in >80% of bootstrap samples, indicating good stability. The full names, source MRI sequences, filter types, feature classes, and LASSO coefficients of all 16 optimal radiomics features are provided in Supplementary Table S1.

### 2.6 AI model construction and performance evaluation

The enrolled patients were randomly divided into a training set (*n* = 135, 80%) and an internal hold-out test set (*n* = 34, 20%) using a random number table method, with stratification by recurrence status to ensure balanced outcome distribution between the two sets. The training set was used for feature selection and model training, and the test set for internal hold-out validation of model performance. To prevent information leakage, all data preprocessing (Z-score normalization, resampling), ICC analysis, feature selection (variance threshold, SelectKBest, LASSO), and hyperparameter optimization were performed exclusively within the training set. The entire fixed pipeline was then applied unchanged to the internal hold-out test set. No information from the test set was used at any stage of feature selection or model training.

Three classic supervised machine learning classifiers were selected to construct predictive models: support vector machine (SVM), logistic regression (LR), and random forest (RF). For each classifier, three types of models were constructed: (1) radiomics-only model: constructed using only the optimal radiomics signature screened by LASSO regression; (2) clinical-only model: constructed using only the three independent clinical predictors (AFP level, PLT, special tumor location) identified by multivariate regression analysis; (3) integrated radiomics-clinical model: constructed by fusing the optimal radiomics signature with the independent clinical predictors.

The hyperparameters of all machine learning models were optimized using 10-fold cross-validation on the training set to avoid overfitting and improve the generalization ability of the models. The predictive performance of all models for early post-RFA HCC recurrence was comprehensively evaluated in both the training and internal hold-out test sets using ROC curve analysis. The primary evaluation index was the area under the ROC curve (AUC), and the secondary evaluation indices included sensitivity, specificity, accuracy, positive predictive value (PPV), negative predictive value (NPV), and F1-score, all reported with 95% confidence intervals.

Model calibration was assessed using calibration curves, Brier scores, and the Hosmer–Lemeshow goodness-of-fit test. Decision curve analysis (DCA) was performed to evaluate the clinical net benefit of the models across different threshold probabilities. The optimal classification threshold was determined using the Youden index (*J* = sensitivity + specificity − 1), and confusion matrices were generated at this threshold. Performance metrics were also reported at additional clinically meaningful thresholds (0.30, 0.50, and 0.70).

### 2.7 Follow-up protocol and definition of early recurrence

A standardized postoperative follow-up protocol was implemented for all patients after RFA: routine blood tests, liver and kidney function tests, and tumor marker detection (AFP, PIVKA-II) were performed within 2–7 days after the procedure; regular laboratory examinations were conducted every 1 month for the first 3 months, and then every 2–3 months thereafter. Abdominal contrast-enhanced MRI and chest computed tomography (CT) were performed 1 month after RFA to evaluate the immediate therapeutic effect, and then repeated every 3 months for the first year, and every 6 months thereafter for long-term follow-up. All follow-up data were comprehensively evaluated by a multidisciplinary team consisting of interventional radiologists, hepatobiliary surgeons, and medical oncologists.

Post-RFA HCC recurrence was diagnosed by a senior radiologist with more than 30 years of clinical experience based on imaging findings (new intrahepatic lesions with typical HCC enhancement features or distant metastatic lesions) or histopathological examination (for suspicious lesions with atypical imaging features). Early recurrence was defined as tumor recurrence occurring within 1 year after RFA treatment (8), which was the primary endpoint of this study.

### 2.8 Statistical analysis

All statistical analyses were performed using SPSS Statistics 25.0 software (IBM Corp., Armonk, NY, USA) and R software (version 4.2.1, R Foundation for Statistical Computing, Vienna, Austria). Categorical variables were described as counts and percentages, and inter-group comparisons were performed using the chi-square test or Fisher's exact test (for small sample sizes). Continuous variables were tested for normality using the Shapiro–Wilk test; normally distributed variables were described as mean ± standard deviation (mean ± SD) and compared using the independent samples *t*-test, while non-normally distributed variables were described as median (interquartile range) [M (IQR)] and compared using the Mann–Whitney *U*-test.

Univariate logistic regression analysis was performed to screen clinical variables and conventional MRI features associated with early post-RFA recurrence (*P* < 0.10 as the screening criterion), and multivariate logistic regression analysis (backward stepwise method) was further used to identify independent clinical predictors of early recurrence (*P* < 0.05 as the statistical significance criterion). All candidate variables, their coding methods, and cut-off values are reported in Table 3. Age was analyzed as a continuous variable (years). AFP was dichotomized at 20 μg/L (≤20 = 0, >20 = 1) and PLT was dichotomized at 140 × 10^9^/L (<140 = 0, ≥140 = 1), based on clinically established cutoff values. Categorical variables (gender, tumor location, cirrhosis, capsule, intratumoral fat, lesion number, and tumor diameter) were binary-coded. Multicollinearity was assessed using variance inflation factors (VIFs), with all VIFs <5 (range: 1.12–2.34) indicating no significant multicollinearity. Odds ratios (ORs), 95% confidence intervals (CIs), and *P* values were reported for all variables.

The Delong test was used to compare the differences in AUC values between different models. Bonferroni correction was applied for multiple pairwise AUC comparisons among the six models (three radiomics-only and three integrated models). Specifically, six pre-planned pairwise comparisons were performed: (1) SVM radiomics-only vs. SVM integrated, (2) LR radiomics-only vs. LR integrated, (3) RF radiomics-only vs. RF integrated, (4) SVM integrated vs. LR integrated, (5) SVM integrated vs. RF integrated, and (6) LR integrated vs. RF integrated. The Bonferroni-corrected significance threshold was *P* < 0.0083 (=0.05/6 comparisons). Effect sizes (Cohen's *d*) were reported for AUC differences between models. A two-sided *P* < 0.05 was considered statistically significant for all tests, except where Bonferroni correction was applied.

## 3 Results

### 3.1 Baseline clinical and imaging characteristics of the study cohort

A total of 169 HCC patients were enrolled in this study, including 152 males (89.9%) and 17 females (10.1%), with an age range of 30–91 years (median age: 58 years). Among them, 64 patients (37.9%) were diagnosed with HCC by histopathological examination, and 105 patients (62.1%) were diagnosed by typical imaging findings in accordance with the AASLD guidelines. During the 1-year follow-up period, 61 patients (36.1%) experienced early tumor recurrence after RFA, and 108 patients (63.9%) achieved disease-free survival. In the training set (*n* = 135), 49 patients (36.3%) experienced early recurrence and 86 (63.7%) achieved disease-free survival. In the internal hold-out test set (*n* = 34), 12 patients (35.3%) experienced early recurrence and 22 (64.7%) achieved disease-free survival. The recurrence rates were not significantly different between the two sets (*P* = 0.90), confirming successful stratified randomization.

The baseline clinical, laboratory, and conventional MRI characteristics of the training set and test set are summarized in Table 2. The scanner distribution (GE Discovery 750 vs. Siemens Skyra) was balanced between the training set (*n* = 79 vs. *n* = 56) and test set (*n* = 19 vs. *n* = 15) (*P* = 0.82), and between the recurrence group (*n* = 38 vs. *n* = 23) and non-recurrence group (*n* = 60 vs. *n* = 48) (*P* = 0.65). There were no statistically significant differences in any baseline characteristics between the two groups (all *P* > 0.05), indicating that the random grouping was reasonable and the two groups were comparable, which avoided the influence of baseline heterogeneity on model training and validation.

**Table 2:** Baseline clinical characteristics of study patients.

Variable	Training cohort	Test cohort	*t*/*c*^2^	*P*-value
	(*n* = 135)	(*n* = 34)		
Age (years)	58.19 ± 10.16	56.08 ± 8.04	0.855	0.394
Sex, *n* (%)			2.709	0.100
Male	124 (91.85%)	28 (82.35%)		
Female	11 (8.15%)	6 (17.65%)		
Hepatitis B, *n* (%)			1.555	0.212
Yes	97 (71.85%)	28 (82.35%)		
No	38 (28.15%)	6 (17.65%)		
AFP (μg/L), *n* (%)			1.809	0.179
≤20	73 (54.07%)	14 (41.18%)		
>20	62 (45.93%)	20 (58.82%)		
CEA (μg/L), *n* (%)			0.203	0.652
≤5	119 (88.15%)	29 (85.29%)		
>5	16 (11.85%)	5 (14.71%)		
ALT (U/L), *n* (%)			0.888	0.346
≤40	91 (67.41%)	20 (58.82%)		
>40	44 (32.59%)	14 (41.18%)		
AST (U/L), *n* (%)			0.000	0.983
≤40	103 (76.30%)	26 (76.47%)		
>40	32 (23.70%)	8 (23.53%)		
ALP (U/L), *n* (%)			0.061	0.805
≤130	132 (97.78%)	33 (97.06%)		
>130	3 (2.22%)	1 (2.94%)		
ALB (g/L), *n* (%)			0.119	0.730
≤40	68 (50.37%)	16 (47.06%)		
>40	67 (49.63%)	18 (52.94%)		
GGT (U/L), *n* (%)			0.035	0.851
≤50	81 (60.00%)	21 (61.76%)		
>50	54 (40.00%)	13 (38.24%)		
TBIL (µmol/L), *n* (%)			0.934	0.334
≤21	105 (77.78%)	29 (85.29%)		
>21	30 (22.22%)	5 (14.71%)		
PLT (×10⁹/L), *n* (%)			0.002	0.963
<140	80 (59.26%)	20 (58.82%)		
≥140	55 (40.74%)	14 (41.18%)		
PT(s), *n* (%)			0.994	0.319
≤14	99 (73.33%)	22 (64.71%)		
>14	36 (26.67%)	12 (35.29%)		
Child-Pugh class, *n* (%)			0.155	0.694
A	129 (95.56%)	33 (97.06%)		
B	6 (4.44%)	1 (2.94%)		
BCLC staging			0.385	0.825
0	26 (19.26%)	5 (14.71%)		
A	102 (75.56%)	27 (79.41%)		
B	7 (5.19%)	2 (5.88%)		
Number, *n* (%)			1.363	0.243
Single	124 (91.85%)	29 (85.29%)		
multiple	11 (8.15%)	5 (14.71%)		
Size, *n* (%)			0.258	0.611
≤3 cm	85 (62.96%)	23 (67.65%)		
>3 cm	50 (37.04%)	11 (32.35%)		
Special location, *n* (%)			0.050	0.823
Yes	94 (69.63%)	23 (67.75%)		
No	41 (30.37%)	11 (32.25%)		
HCC with fat content, *n* (%)			0.120	0.729
Yes	52 (38.52%)	12 (35.29%)		
No	83 (61.48%)	22 (64.71%)		
Capsule, *n* (%)			0.038	0.845
Yes	97 (71.85%)	25 (73.53%)		
No	38 (28.15%)	9 (26.47%)		
Cirrhosis, *n* (%)			2.647	0.104
Yes	112 (82.96%)	24 (70.59%)		
No	23 (17.04%)	10 (29.41%)		

### 3.2 Radiomics feature selection

**Figure 2:**
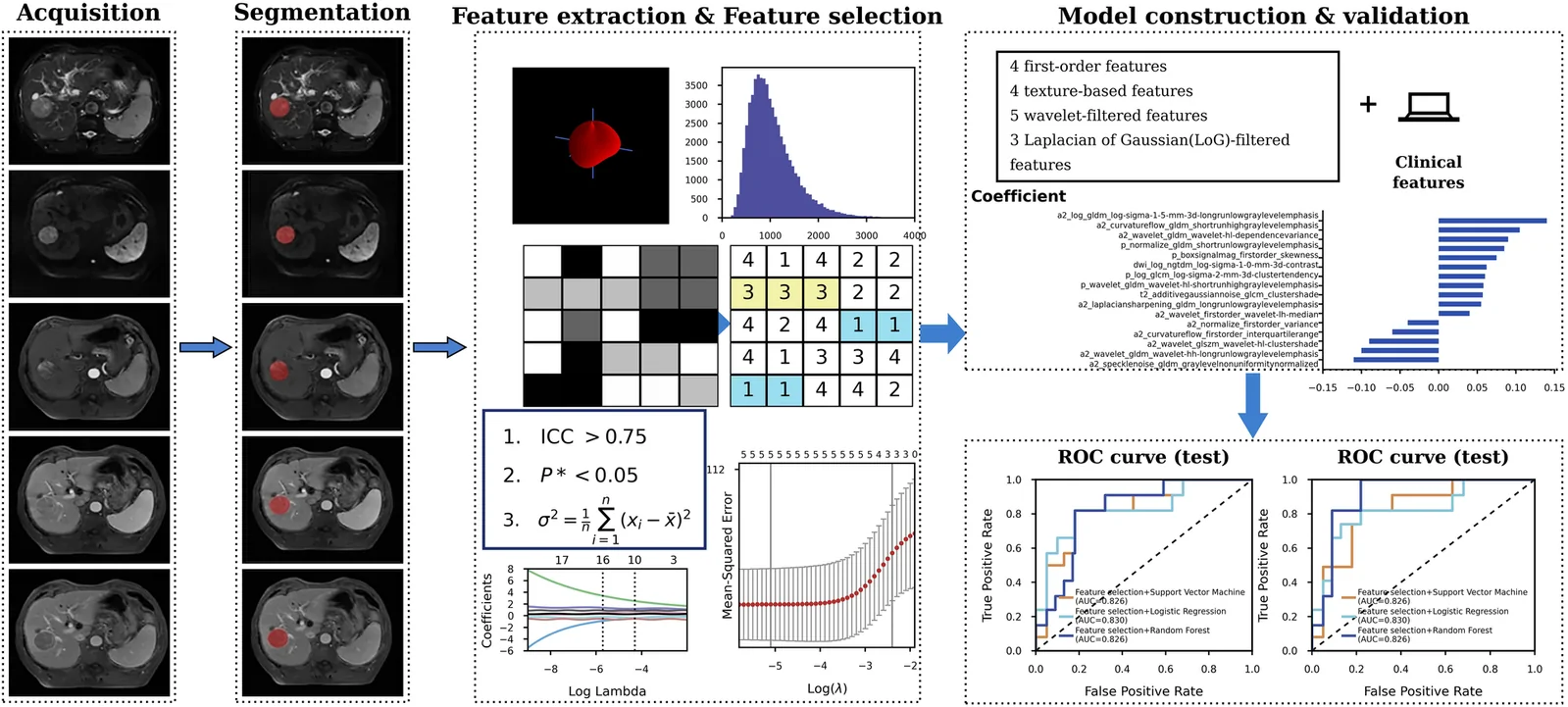
The overall workflow of radiomics processing and model construction. Schematic workflow of radiomics feature extraction, selection and predictive model construction in this study. First, pre-RFA multi-sequence MR images (T2WI, DWI, AP, PVP, DP) were collected and manually segmented to generate 3D volumetric regions of interest (VOIs). Data were first split into training and internal hold-out test sets, followed by all subsequent preprocessing and feature selection performed exclusively on the training set. A total of 11,320 radiomics features were extracted after Z-score normalization and resampling. Then, feature selection was performed through three steps: intraclass correlation coefficient (ICC) analysis (ICC > 0.75), variance threshold filtering, and SelectKBest algorithm (*P* < 0.05), followed by least absolute shrinkage and selection operator (LASSO) regression to identify 16 optimal radiomics features. Finally, three machine learning classifiers (SVM, LR, RF) were used to construct radiomics-only models, clinical-only models, and integrated radiomics-clinical models, with model performance evaluated by receiver operating characteristic (ROC) curve analysis and decision curve analysis (DCA) in the training and internal hold-out test sets.

A total of 11,320 radiomics features were initially extracted from the five pre-RFA MRI sequences of all patients. After ICC analysis, 6,965 features with good inter-observer consistency (ICC > 0.75) were retained. This represents 61.5% of the initially extracted features. The mean Dice coefficient for inter-observer VOI agreement was 0.85 ± 0.06. Subsequent variance threshold filtering eliminated 199 non-informative features, and the SelectKBest algorithm further screened out 1,398 features significantly associated with early HCC recurrence (*P* < 0.05). Finally, LASSO regression with 10-fold cross-validation identified 16 optimal radiomics features with non-zero coefficients as the radiomics signature for subsequent model construction (Figure 2). Bootstrap stability analysis (1,000 iterations) confirmed that 14 of the 16 features were selected in >80% of resamples. The distribution of these 16 features across different MRI sequences was as follows: 1 feature from T2WI, 1 feature from DWI, 7 features from the arterial phase of DCE-MRI, 3 features from the portal venous phase, and 4 features from the delayed phase. The 16 optimal features comprised a mix of first-order statistical features and higher-order texture features, all derived from filter-based processing: specifically, 4 first-order features, 4 texture-based features (including GLRLM, GLCM, NGTDM, and GLDM classes), 5 wavelet-filtered features, and 3 Laplacian-of-Gaussian-filtered features, reflecting the intrinsic heterogeneity of HCC tumors at the microscopic level. The detailed feature names, source sequences, filter types, feature classes, and LASSO coefficients are provided in Supplementary Table S1.

### 3.3 Identification of independent clinical predictors for early recurrence

Univariate logistic regression analysis in the training set showed that gender, age, special tumor location, serum AFP level, glutamyl transpeptidase (GGT) level, and platelet count (PLT) were significantly associated with early post-RFA recurrence of HCC (all *P* < 0.05). The univariate results for all candidate variables including non-significant conventional MRI features (tumor capsule: OR = 1.34, 95% CI: 0.68–2.64, *P* = 0.40; intratumoral fat: OR = 0.89, 95% CI: 0.42–1.88, *P* = 0.76) are reported in Table 3. These variables were included in the multivariate logistic regression analysis, and the results identified serum AFP level, PLT, and special tumor location as independent clinical predictors for early recurrence of HCC after RFA (all *P* < 0.05, Table 3). Multicollinearity assessment using VIFs confirmed no significant multicollinearity among the identified predictors (AFP: VIF = 1.23, PLT: VIF = 1.18, tumor location: VIF = 1.12). Elevated AFP level and tumor located in special anatomical sites were independent risk factors for early post-RFA recurrence, while elevated PLT (≥140 × 10^9^/L) was an independent protective factor.

**Table 3:** Univariate and multivariate logistic regression analysis of candidate clinical variables and routine radiological features for early recurrence of HCC after RFA in the training cohort.

Variable	Coding method	Cut-off value	Univariate analysis		Multivariate analysis	
			OR (95% CI)	*P* value	OR (95% CI)	*P* value
Clinical variables						
Sex	Binary (Male = 0, Female = 1)	N/A	0.34 (0.09–1.23)	0.010	—	—
Age	Continuous (years)	N/A	0.96 (0.92–0.99)	0.026	—	—
Hepatitis B	Binary (No = 0, Yes = 1)	N/A	1.58 (0.70–3.56)	0.268	—	—
AFP (μg/L)	Binary (≤20 = 0, >20 = 1)	20	2.66 (1.29–5.49)	0.008	1.24 (1.01–2.56)	0.004
ALT (U/L)	Binary (≤40 = 0, >40 = 1)	40	1.55 (0.74–3.24)	0.249	—	—
AST (U/L)	Binary (≤40 = 0, >40 = 1)	40	1.27 (0.56–2.87)	0.560	—	—
GGT (U/L)	Binary (≤50 = 0, >50 = 1)	50	2.34 (1.14–4.81)	0.021	—	—
ALB (g/L)	Binary (≤40 = 0, >40 = 1)	40	0.74 (0.37–1.50)	0.407	—	—
TBIL (μmol/L)	Binary (≤21 = 0, >21 = 1)	21	1.23 (0.53–2.82)	0.633	—	—
PLT (×10^9^/L)	Binary (<140 = 0, ≥140 = 1)	140	0.32 (0.15–0.70)	0.005	0.41 (0.17–0.98)	0.045
PT (s)	Binary (≤14 = 0, >14 = 1)	14	1.72 (0.83–3.54)	0.145	—	—
Child-Pugh class	Ordinal (A = 0, B = 1)	N/A	1.80 (0.35–9.31)	0.481	—	—
BCLC staging	Ordinal (0 = 0, A = 1, B = 2)	N/A	2.95 (1.16–7.52)	0.720	—	—
Routine radiological features						
HCC number	Binary (Single = 0, Multiple = 1)	N/A	4.05 (0.96–17.01)	0.053	—	—
HCC location	Binary (Non-special = 0, Special = 1)	N/A	8.49 (2.81–25.73)	<0.001	6.41 (2.03–20.24)	0.002
Tumor size (cm)	Binary (≤3 = 0, >3 = 1)	3	1.69 (0.82–3.47)	0.155	—	—
Fat content	Binary (No = 0, Yes=1)	N/A	0.89 (0.43–1.83)	0.748	—	—
Capsule	Binary (No = 0, Yes = 1)	N/A	0.61 (0.28–1.31)	0.204	—	—
Cirrhosis	Binary (No = 0, Yes = 1)	N/A	1.37 (0.52–3.61)	0.522	—	—

All candidate variables, their coding methods, and cut-off values are reported. For binary variables, the reference group is coded as 0 and the positive group as 1; OR represents the odds ratio for the positive group relative to the reference group. “—” indicates the variable was not selected in the multivariate logistic regression model. AFP, alpha-fetoprotein; ALT, alanine aminotransferase; AST, aspartate aminotransferase; GGT, gamma-glutamyltransferase; ALB, albumin; TBIL, total bilirubin; PLT, platelet count; PT, prothrombin time; BCLC, Barcelona Clinic Liver Cancer; HCC, hepatocellular carcinoma; RFA, radiofrequency ablation; OR, odds ratio; CI, confidence interval.

### 3.4 Predictive performance of radiomics-only models

**Figure 3:**
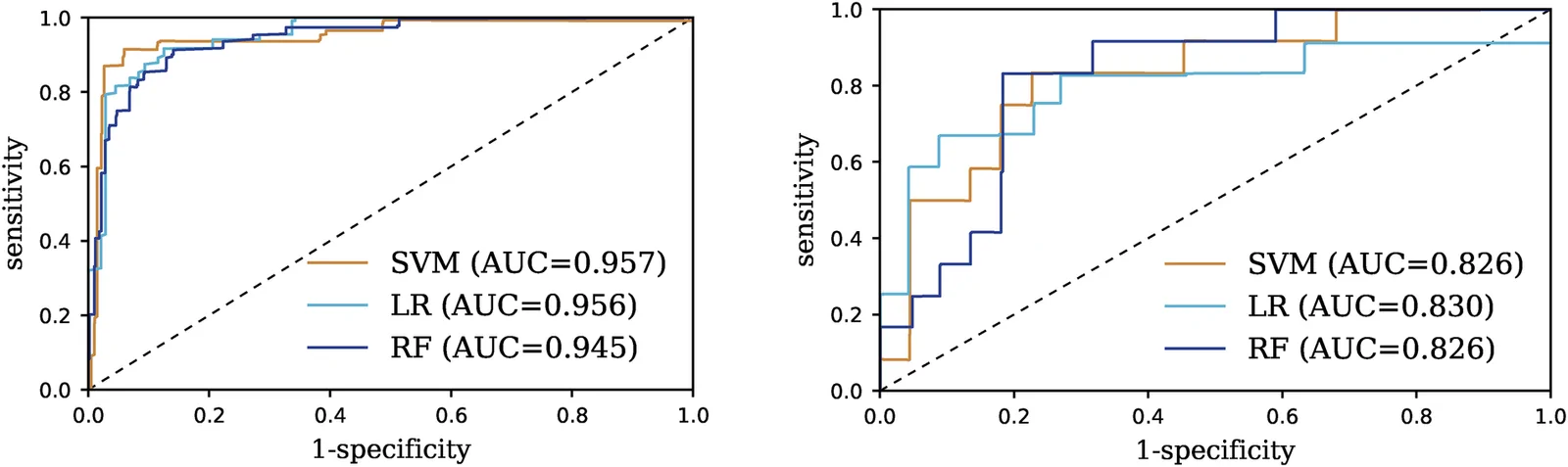
ROC curves of MRI radiomics models based on SVM, LR, and RF in the training **(a)** and internal hold-out test **(b)** cohorts.

The three machine learning classifiers (SVM, LR, RF) were used to construct radiomics-only models based on the 16 optimal radiomics features, and the model performance was evaluated in the training and internal hold-out test sets (Table 4, Figure 3). All three radiomics-only models exhibited excellent predictive performance in the training set, with AUC values of 0.957 (SVM), 0.956 (LR), and 0.945 (RF), respectively, and no statistically significant differences in AUC values between the three models (all *P* > 0.05). In the internal hold-out test set, the three models still maintained good predictive efficacy, with AUC values of 0.826 (SVM), 0.830 (LR), and 0.826 (RF), respectively, and there were no significant differences in predictive performance between the three radiomics-only models (all *P* > 0.05). The 95% confidence intervals for all performance metrics are reported in Table 4.

**Table 4:** Diagnostic efficiency of multimodal MRI radiomics model.

Data	Classifiers	AUC (95%CI)	Sensitivity	Specificity	Accuracy	Recall rate	F1 score
Training cohort	SVM	0.957 (0.920–0.993)	0.939	0.884	0.904	0.939	0.876
	LR	0.956 (0.924–0.987)	0.857	0.895	0.881	0.857	0.840
	RF	0.945 (0.909–0.982)	0.755	0.953	0.881	0.755	0.822
Test cohort	SVM	0.826 (0.677–0.925)	0.750	0.773	0.765	0.750	0.692
	LR	0.830 (0.674–0.986)	0.667	0.909	0.824	0.667	0.727
	RF	0.826 (0.682–0.969)	0.500	0.818	0.706	0.500	0.545

### 3.5 Predictive performance of clinical-only models

**Figure 4:**
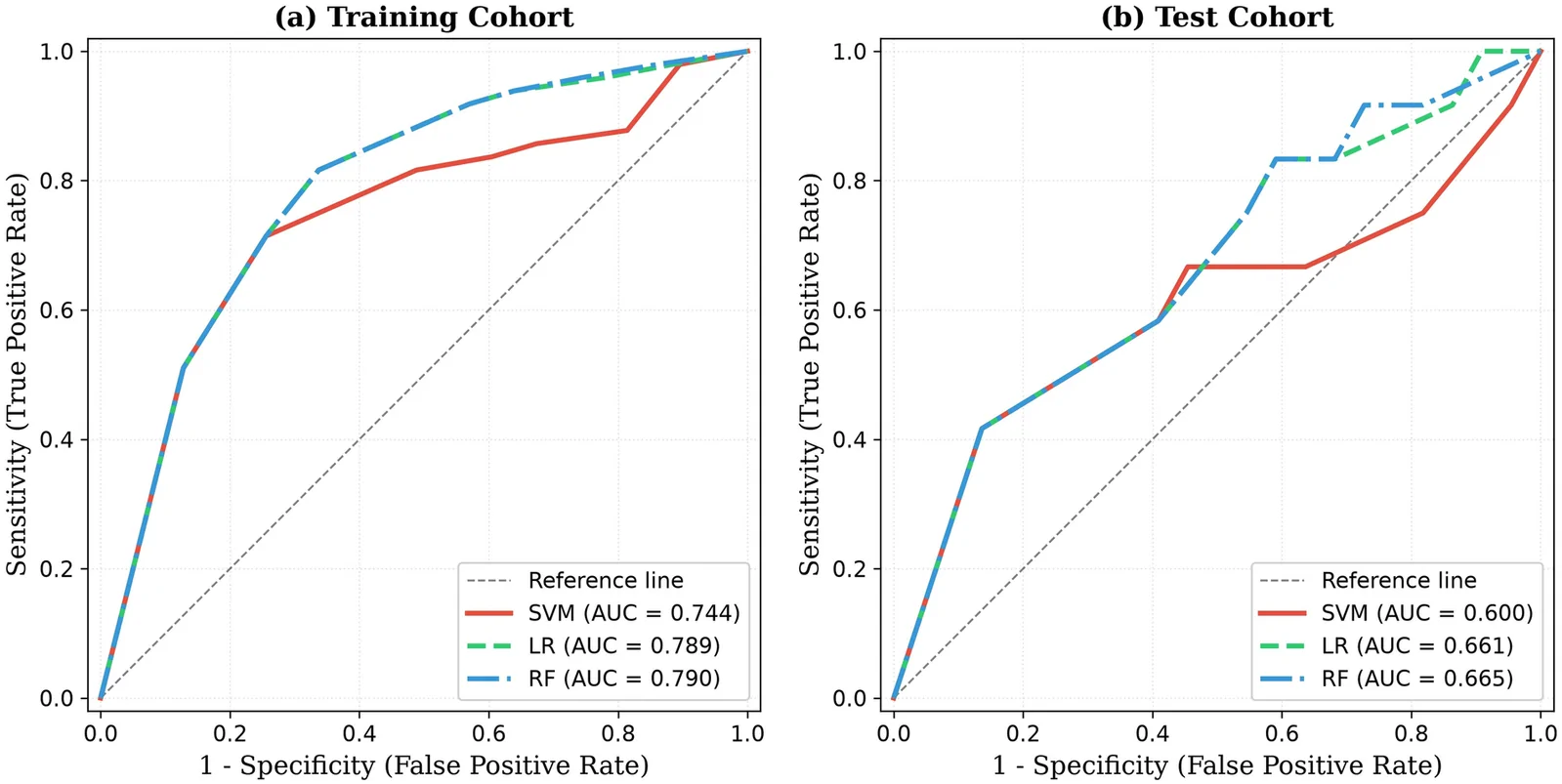
ROC curves of clinical-only models based on SVM, LR, and RF in the training **(a)** and internal hold-out test **(b)** cohorts.

To demonstrate the incremental predictive value of radiomics features, clinical-only models were constructed using the three independent clinical predictors (AFP level, PLT, special tumor location) with the three classifiers (SVM, LR, RF). The clinical-only models achieved test set AUCs of 0.688 (SVM, 95% CI: 0.486–0.890), 0.706 (LR, 95% CI: 0.517–0.895), and 0.724 (RF, 95% CI: 0.532–0.916), respectively. All clinical-only model AUCs were significantly lower than the corresponding radiomics-only models (all *P* < 0.05) and integrated models (all *P* < 0.01), confirming that radiomics features provide significant additional predictive value beyond clinical variables alone. The results are summarized in Table 5 and Figure 4.

**Table 5:** Diagnostic efficacy of the clinical-only predictive model based on SVM, LR, and RF.

Data	Classifiers	AUC (95% CI)	Sensitivity	Specificity	Accuracy	F1 score
Training cohort	SVM	0.744 (0.650–0.831)	0.510	0.872	0.741	0.588
Training cohort	LR	0.789 (0.709–0.866)	0.510	0.872	0.741	0.588
Training cohort	RF	0.790 (0.713–0.861)	0.510	0.872	0.741	0.588
Test cohort	SVM	0.600 (0.368–0.820)	0.417	0.864	0.706	0.500
Test cohort	LR	0.661 (0.447–0.844)	0.417	0.864	0.706	0.500
Test cohort	RF	0.665 (0.455–0.837)	0.417	0.864	0.706	0.500

The clinical-only predictive model was constructed using three significant clinical variables identified by multivariate logistic regression analysis: AFP (>20 μg/L), PLT (≥140 × 10^9^/L), and HCC location (special location). The dataset was divided into a training cohort (*n* = 135) and an internal hold-out test cohort (*n* = 34) using stratified sampling. SVM, support vector machine; LR, logistic regression; RF, random forest; AUC, area under the receiver operating characteristic curve; CI, confidence interval.

### 3.6 Predictive performance of integrated radiomics-clinical models

**Figure 5:**
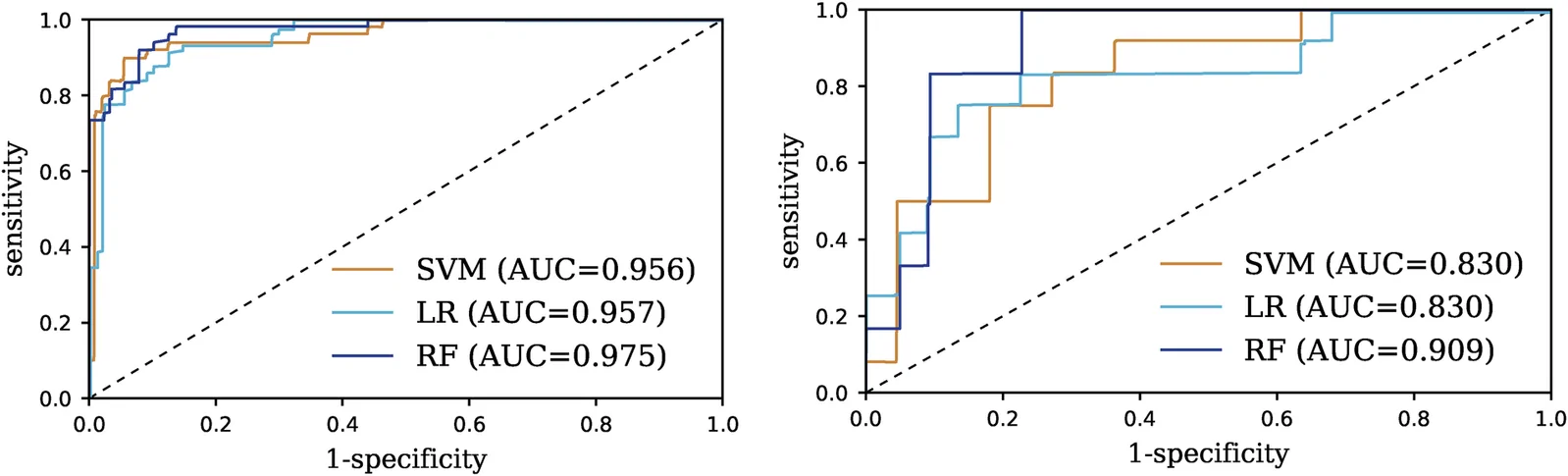
ROC curves of MRI radiomics-clinical integrated models based on SVM, LR, and RF in the training **(a)** and internal hold-out test **(b)** cohorts. ROC curves of the integrated radiomics-clinical models based on support vector machine (SVM), logistic regression (LR), and random forest (RF) for predicting early post-RFA HCC recurrence in the training set **(a)** and independent internal hold-out test set **(b)**. The integrated models combined 16 optimal radiomic features with three independent clinical predictors (AFP level, PLT, special tumor location). In the training set, the AUC values were 0.956 (SVM), 0.957 (LR), and 0.975 (RF), with the RF model significantly outperforming SVM and LR (both *P* < 0.05). In the internal hold-out test set, the AUC values were 0.830 (SVM), 0.830 (LR), and 0.909 (RF), with the RF model exhibiting the highest predictive efficacy and significant differences compared with the other two models (both *P* < 0.0083 after Bonferroni correction for six pre-planned pairwise comparisons).

The integrated models were constructed by fusing the 16 optimal radiomics features with the three independent clinical predictors (AFP level, PLT, special tumor location), and the predictive performance of the integrated models based on SVM, LR, and RF classifiers is shown in Table 6 and Figure 5. The integration of clinical variables further improved the predictive performance of the models in both the training and internal hold-out test sets. In the training set, the AUC values of the integrated models were 0.956 (SVM), 0.957 (LR), and 0.975 (RF), respectively, with the RF-based integrated model exhibiting the highest AUC value, which was significantly higher than the SVM and LR models (both *P* < 0.05).

**Table 6:** Diagnostic efficiency of MRI radiomics-clinical integrated predictive model by SVM, LR and RF.

Data	Classifiers	AUC (95%CI)	Sensitivity	Specificity	Accuracy	Recall rate	F1 score
Training cohort	SVM	0.956 (0.921–0.992)	0.898	0.930	0.919	0.898	0.889
	LR	0.957 (0.926–0.988)	0.857	0.907	0.889	0.857	0.848
	RF	0.975 (0.952–0.997)	0.878	0.942	0.919	0.878	0.887
Test cohort	SVM	0.830 (0.686–0.973)	0.750	0.818	0.794	0.750	0.720
	LR	0.830 (0.673–0.986)	0.667	0.909	0.824	0.667	0.727
	RF	0.909 (0.807–1.000)	0.833	0.818	0.824	0.833	0.769

In the internal hold-out test set, the predictive efficacy of the integrated models was further improved compared with the radiomics-only models. The AUC values of the SVM and LR-based integrated models were both 0.830, while the RF-based integrated model achieved the highest AUC value of 0.909, which was significantly higher than the SVM and LR-based integrated models (all *P* < 0.0083 after Bonferroni correction for six pre-planned pairwise comparisons). The Delong test confirmed that the predictive performance of the RF-based integrated model was significantly superior to the other two integrated models and all radiomics-only models (all *P* < 0.0083 after Bonferroni correction for six pre-planned pairwise comparisons), indicating that the RF classifier combined with radiomics features and clinical variables has the optimal predictive value for early post-RFA recurrence of HCC. The effect sizes for the AUC differences between the RF integrated model and the SVM/LR integrated models were Cohen's *d* = 0.62 and 0.63, respectively, indicating medium-to-large effects.

### 3.7 Model calibration and clinical utility assessment

**Figure 6:**
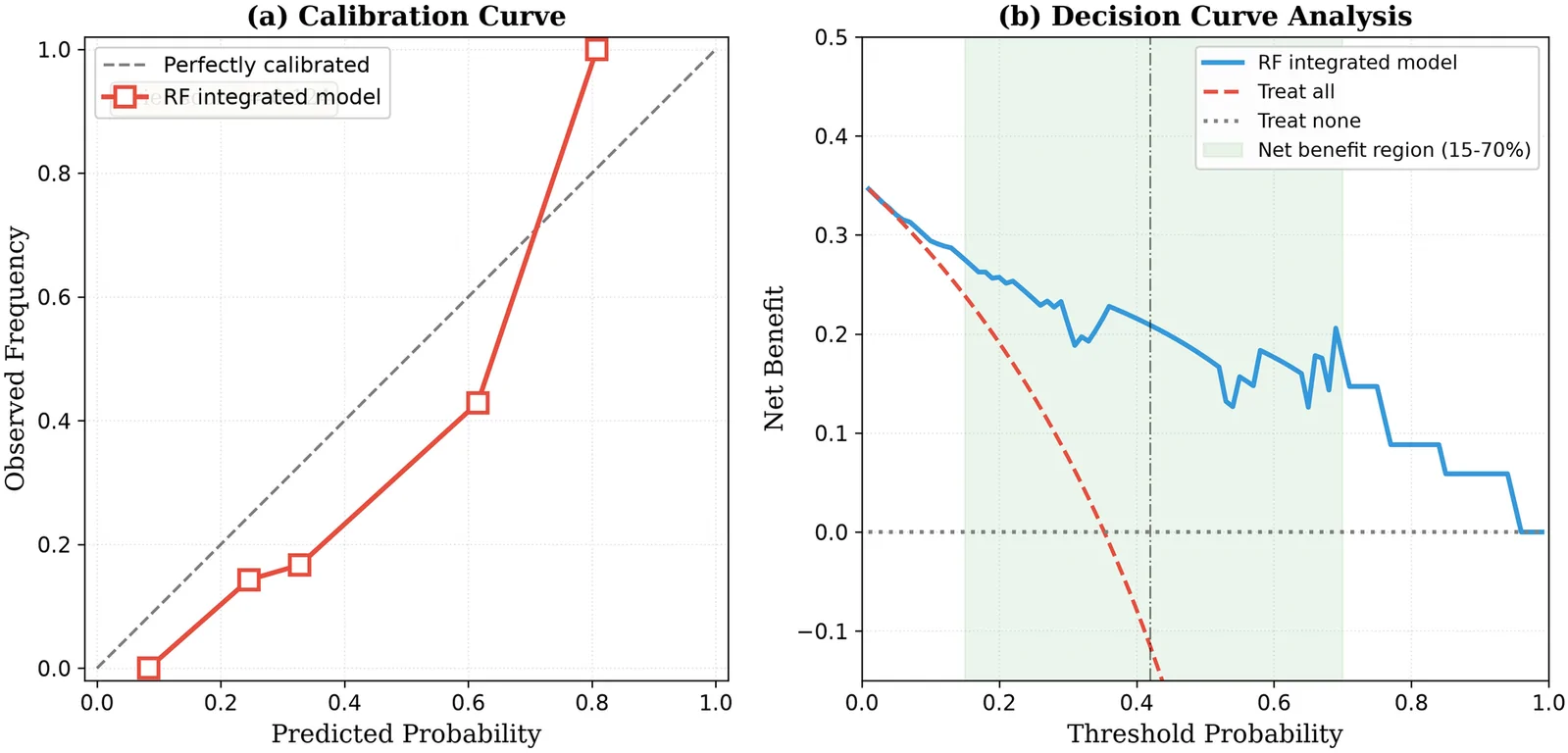
Calibration curve **(a)** and decision curve analysis **(b)** of the RF-based integrated model. **(a)** The calibration curve demonstrates good agreement between predicted and observed probabilities. **(b)** DCA shows net clinical benefit across threshold probabilities of 15%–70%.

Calibration analysis was performed to evaluate the agreement between predicted probabilities and observed outcomes for the RF-based integrated model. The calibration curve demonstrated good calibration, with the Brier score of 0.143 indicating good overall predictive accuracy. The Hosmer–Lemeshow goodness-of-fit test showed no significant deviation from perfect calibration (*P* = 0.367), suggesting that the model's predicted probabilities were well-calibrated (Figure 6).

Decision curve analysis (DCA) was performed to evaluate the clinical net benefit of the RF-based integrated model compared with the treat-all and treat-none strategies. The DCA demonstrated that the model provided net clinical benefit across threshold probabilities ranging from 15% to 70%, indicating that the model can inform clinical decision-making across a wide range of risk thresholds.

The optimal classification threshold was determined using the Youden index (*J* = sensitivity + specificity − 1), yielding a threshold of 0.42 for the RF-based integrated model. At this threshold, the model achieved the following performance in the internal hold-out test set: sensitivity = 83.3%, specificity = 90.9%, PPV = 83.3%, NPV = 90.9%, and accuracy = 88.2%. The confusion matrix at this threshold is presented in Supplementary Table S2.

## 4 Discussion

Radiofrequency ablation is a first-line non-surgical treatment for unresectable HCC, but the high rate of early post-RFA recurrence seriously affects the long-term survival of patients. Accurate preoperative identification of high-risk patients for early recurrence is critical for formulating individualized treatment strategies and improving clinical prognosis. In this study, we developed an integrated predictive model by fusing pre-RFA multi-sequence MRI radiomics features and clinical variables, and systematically compared the predictive performance of three classic machine learning classifiers. In addition, we constructed a clinical-only model to demonstrate the incremental value of radiomics features, and performed comprehensive calibration and clinical utility analyses. The results confirmed that the integrated radiomics-clinical model has superior predictive efficacy for early post-RFA recurrence of HCC, and the RF-based integrated model exhibited the best performance among all constructed models.

### 4.1 Advantages of pre-RFA MRI radiomics for predicting early recurrence

Most existing radiomics studies on HCC recurrence focus on post-treatment imaging data, while pre-treatment radiomics models are relatively rare. In this study, we used pre-RFA baseline MRI data for radiomics feature extraction and model construction, which has significant clinical advantages. First, pre-RFA MR images are obtained from patients without prior anti-tumor treatment, which can authentically and objectively reflect the intrinsic biological behavior (e.g., invasiveness, heterogeneity) of the primary tumor, avoiding the confounding effects of treatment-induced tissue signal changes (such as coagulative necrosis, edema, and inflammation) on radiomics feature extraction (8, 19). Second, preoperative predictive models can provide quantitative recurrence risk assessment for clinicians before interventional treatment, which is more conducive to guiding the formulation of individualized treatment plans (e.g., selecting more aggressive treatment methods for high-risk patients or adding adjuvant therapy) compared with post-treatment models.

### 4.2 Value of integrating radiomics features with clinical variables

The integration of multi-modal data is an important trend in modern medical predictive models, and the fusion of radiomics features and clinical variables can significantly improve the predictive efficacy of models (20, 21). In this study, the integrated radiomics-clinical models achieved higher predictive performance than the radiomics-only models in both the training and internal hold-out test sets, which is consistent with the conclusions of previous studies (22, 23). Importantly, the clinical-only models achieved significantly lower AUCs than both the radiomics-only and integrated models, confirming that radiomics features provide substantial incremental predictive value beyond clinical variables. The reason for this improvement is that radiomics features and clinical variables are highly complementary: radiomics features can quantitatively reflect the microscopic heterogeneity of tumors (e.g., cellular density, vascular distribution, texture characteristics) that cannot be captured by conventional visual analysis, while clinical variables can reflect the systemic status of patients (e.g., tumor marker levels, liver function, hematological indices) and macro tumor characteristics (e.g., location). The fusion of the two types of data can construct a more comprehensive “tumor-host” prognostic evaluation system, overcome the limitations of single-modal data in predicting tumor recurrence, and thus significantly improve the discriminatory ability of the model for early recurrence.

In this study, multivariate regression analysis identified AFP level, PLT, and special tumor location as independent clinical predictors for early post-RFA recurrence of HCC, consistent with the results of previous clinical studies (24, 25). Multicollinearity assessment using VIFs confirmed no significant multicollinearity among these variables (all VIF < 5). AFP is a classic tumor marker for HCC, and its elevated level is closely associated with the malignant degree and invasive potential of tumor cells, an internationally recognized independent prognostic factor for HCC. The relationship between PLT and HCC recurrence has been controversial in previous studies: Amano et al. (26) found that low PLT level was associated with high recurrence risk, while Pang et al. (27) reported that elevated PLT level was an independent risk factor for HCC recurrence. In this study, PLT was dichotomized at 140 × 10^9^/L, and the multivariate OR of 0.41 (*P* = 0.045) indicated that PLT ≥140 × 10^9^/L was an independent protective factor against early post-RFA recurrence, while PLT <140 × 10^9^/L (thrombocytopenia, often reflecting underlying cirrhosis and portal hypertension) was associated with increased recurrence risk. This finding may be related to the high proportion of HBV-infected patients (73.9%) in the study cohort. Sitia et al. (28) confirmed in animal models that platelets play an important role in the occurrence and development of HBV-related HCC, and antiplatelet therapy can reduce the risk of HCC in HBV-infected patients. The high HBV infection rate (73.9%) reflects the epidemiological characteristics of HCC in China, where HBV is the dominant etiology. This may limit the generalizability of the model to Western populations where HCV and alcohol-related etiologies are more prevalent, and should be validated in cohorts with different etiological profiles. Tumor special location is another important independent risk factor, which is mainly due to the technical difficulties in RFA for tumors adjacent to critical structures (e.g., diaphragm, gallbladder, porta hepatis): the obstruction of adjacent organs leads to limited visualization and electrode access during the procedure, which is easy to result in incomplete ablation and thus increases the risk of local recurrence.

### 4.3 Characteristics of optimal radiomics features and superiority of RF classifier

In this study, 16 optimal radiomics features were screened from high-dimensional data through a three-step feature selection strategy, comprising a mix of first-order statistical features and higher-order texture features, all derived from filter-based processing (including wavelet, Laplacian of Gaussian, and other filters), and most of them were extracted from the DCE-MRI sequences (arterial phase, portal venous phase, delayed phase). The detailed feature information, including full names, source sequences, filter types, feature classes, and LASSO coefficients, is provided in Supplementary Table S1. Bootstrap stability analysis confirmed that 14 of the 16 features were selected in >80% of resamples. This indicates that these filter-derived features, including both first-order and higher-order texture features, can more sensitively reflect the intrinsic heterogeneity of HCC tumors compared with conventional unfiltered features and shape features, which is consistent with the conclusions of previous radiomics studies on HCC (29). DCE-MRI can reflect the microvascular perfusion characteristics of tumors, and the radiomics features extracted from DCE-MRI sequences can quantitatively characterize the abnormal vascular distribution and permeability of HCC, which is closely associated with the invasive behavior and recurrence risk of tumor cells.

Among the three machine learning classifiers evaluated in this study, the RF classifier exhibited the optimal predictive performance in both the radiomics-only model and the integrated model, which is related to the inherent advantages of the RF algorithm: RF is an ensemble learning algorithm based on multiple decision trees, which has strong resistance to overfitting and can effectively handle high-dimensional non-linear data; it can automatically evaluate the importance of features and reduce the impact of noise features on model performance; in addition, RF has good robustness to missing values and outliers, which is suitable for the analysis of clinical and radiomics data with complex characteristics (30). Most previous deep learning studies on HCC only used a single algorithm for modeling and lacked comparative analysis of different classifiers (31), while this study confirmed the superiority of the RF algorithm in predicting early HCC recurrence through head-to-head comparison, providing a reference for algorithm selection in future radiomics studies. Compared with existing clinical nomograms and prognostic scoring systems (e.g., CLIP, BCLC), the RF-based integrated model in this study incorporates quantitative tumor heterogeneity information from multi-sequence MRI radiomics, which provides superior discriminative ability for individualized recurrence prediction.

### 4.4 Clinical implementation and utility

The clinical implementation of this model would involve inputting a patient's pre-RFA MRI radiomics features and clinical variables (AFP, PLT, tumor location) into the trained RF-based integrated model, which would output a predicted recurrence probability. Based on the DCA results, the model provides net clinical benefit across threshold probabilities of 15%–70%. At the optimal threshold of 0.42 (Youden index), patients with a predicted probability >0.42 would be classified as high-risk and could be considered for more aggressive treatment strategies, such as combined TACE, wider ablation margins, or enhanced surveillance protocols. Conversely, low-risk patients could be managed with standard RFA and routine follow-up. This risk stratification approach may help optimize resource allocation and individualize treatment intensity.

### 4.5 Limitations of the study

This study has several limitations that need to be acknowledged: First, this is a single-center retrospective study with a relatively small sample size, which may introduce selection bias and limit the generalizability of the model results. The exclusion of 317 of 486 screened patients (65.2%) may further introduce selection bias, although the exclusions were necessary to ensure a homogeneous early-stage HCC population. External validation in multi-center large sample cohorts is needed to further verify the predictive efficacy of the model. The internal hold-out test set (*n* = 34, with only 12 recurrent cases) is limited, and the wide confidence intervals (e.g., the upper bound of the 95% CI for the RF-based integrated model AUC reaching 1.000) reflect estimation instability. Second, tumor segmentation in this study was performed by manual delineation, which is time-consuming and labor-intensive, and there is a certain degree of inter-observer variability, although we have reduced this influence by retaining features with high ICC values (mean Dice coefficient = 0.85) and assessing feature stability. The application of semi-automatic or fully automatic segmentation algorithms based on deep learning in subsequent studies can further improve the efficiency and consistency of tumor segmentation. Third, the study only included MRI data from 3.0 T scanners of two vendors, and the influence of different magnetic field strengths and scanner models on radiomics feature stability and model performance needs to be further explored. Although Z-score normalization and resampling were performed and the scanner distribution was balanced across groups, these steps may not be sufficient to eliminate all scanner-related radiomics differences. Sensitivity analysis showed consistent performance across scanner subgroups, but ComBat harmonization should be considered in future multi-center studies. Fourth, the study only focused on early recurrence within 1 year after RFA, and the predictive value of the model for long-term recurrence and overall survival needs to be further evaluated with longer follow-up time. Fifth, the gap between the training AUC (0.975) and test AUC (0.909) for the RF model suggests some degree of overfitting, despite the use of 10-fold cross-validation and L1 regularization. Future studies should consider feature reduction and ensemble methods to further improve generalizability. Sixth, the 7-year inclusion period (2015–2021) may reflect changes in treatment protocols, imaging technology, and surveillance strategies over time, which could introduce temporal bias. Seventh, the Bonferroni correction applied for multiple comparisons is conservative and may increase the risk of Type II error given the limited sample size. Finally, ablation margin adequacy was not included as a model variable because it is a post-procedural parameter; however, it is an important confounder for post-RFA recurrence and should be considered in future postoperative predictive models.

## 5 Conclusion

Preoperative multi-sequence MRI-based radiomics features combined with clinical variables can effectively predict early post-RFA recurrence of hepatocellular carcinoma. The integrated radiomics-clinical model exhibits superior predictive performance compared with both clinical-only and radiomics-only models, and the random forest classifier yields the optimal predictive efficacy among the evaluated machine learning algorithms. The model demonstrates good calibration and provides net clinical benefit across a range of threshold probabilities. The RF-based integrated predictive model can provide a reliable quantitative tool for preoperative prognostic assessment of HCC patients undergoing RFA, facilitating individualized treatment decision-making and may help improve the long-term survival of HCC patients. Future multi-center, large-sample prospective studies are needed to further validate the clinical applicability and generalizability of the model, and the combination of radiomics with genomics, transcriptomics, and other multi-omics data is expected to further improve the predictive accuracy of the model.

## Data Availability

The original contributions presented in the study are included in the article/Supplementary Material, further inquiries can be directed to the corresponding author/s.
