# Supplementary material for: Predictive value of an AI model integrating MRI radiomics and clinical features for early recurrence of hepatocellular carcinoma prior to radiofrequency ablation
Source: Front Radiol. 2026 Aug 18;6:1824091. doi: 10.3389/fradi.2026.1824091 (PMC13530903; doi:10.3389/fradi.2026.1824091)
Supplement: Supplementary file 1 [file Supplementaryfile1.docx]

# Supplementary Table S1

Detailed Information of the 16 Optimal Radiomics Features

| **No.** | **Full Feature Name** | **Source MRI Sequence** | **Filter Type** | **Feature Class** | **LASSO Coefficient** |
| --- | --- | --- | --- | --- | --- |
| 1 | v2_specklenoise_glrlm_graylevelnonuniformitynormalized | Portal venous phase (DCE-MRI) | Speckle noise | GLRLM | 0.1398124100 |
| 2 | v2_wavelet_glrlm_wavelet-lhl-longrunlowgraylevelemphasis | Portal venous phase (DCE-MRI) | Wavelet-LHL | GLRLM | 0.1045984180 |
| 3 | a2_wavelet_glcm_wavelet-lhl-clustershade | Arterial phase (DCE-MRI) | Wavelet-LHL | GLCM | 0.0920003057 |
| 4 | v2_curvatureflow_firstorder_interquartilerange | Portal venous phase (DCE-MRI) | Curvature flow | First-order | 0.0877837900 |
| 5 | a2_normalize_firstorder_variance | Arterial phase (DCE-MRI) | Normalize | First-order | 0.0806145300 |
| 6 | a2_wavelet_firstorder_wavelet-lhh-median | Arterial phase (DCE-MRI) | Wavelet-LHH | First-order | 0.0648694262 |
| 7 | a2_laplaciansharpening_glrlm_longrunlowgraylevelemphasis | Arterial phase (DCE-MRI) | Laplacian sharpening | GLRLM | 0.0621786863 |
| 8 | t2_additivegaussiannoise_glcm_clustershade | T2WI | Additive Gaussian noise | GLCM | 0.0614414900 |
| 9 | p_wavelet_glrlm_wavelet-hll-shortrunhighgraylevelemphasis | Delayed phase (DCE-MRI) | Wavelet-HLL | GLRLM | 0.0613962747 |
| 10 | p_log_glcm_log-sigma-2-mm-3d-clustertendency | Delayed phase (DCE-MRI) | LoG (σ=2.0 mm, 3D) | GLCM | 0.0570187500 |
| 11 | dwi_log_ngtdm_log-sigma-1-0-mm-3d-contrast | DWI (b=800) | LoG (σ=1.0 mm, 3D) | NGTDM | 0.0418125900 |
| 12 | p_boxsigmaimage_firstorder_skewness | Delayed phase (DCE-MRI) | Box sigma image | First-order | -0.0411308371 |
| 13 | p_normalize_glrlm_shortrunhighgraylevelemphasis | Delayed phase (DCE-MRI) | Normalize | GLRLM | -0.0638349600 |
| 14 | a2_wavelet_gldm_wavelet-hll-dependencevariance | Arterial phase (DCE-MRI) | Wavelet-HLL | GLDM | -0.0866832957 |
| 15 | a2_curvatureflow_glrlm_shortrunhighgraylevelemphasis | Arterial phase (DCE-MRI) | Curvature flow | GLRLM | -0.0994288200 |
| 16 | a2_log_glrlm_log-sigma-1-5-mm-3d-longrunlowgraylevelemphasis | Arterial phase (DCE-MRI) | LoG (σ=1.5 mm, 3D) | GLRLM | -0.1129302160 |

**Note:** The 16 optimal radiomics features were selected by LASSO regression from the training set after variance threshold filtering and SelectKBest selection. All features are high-order transformation features derived from filter-based processing. Source sequence abbreviations: a2 = arterial phase (DCE-MRI); v2 = portal venous phase (DCE-MRI); p = delayed phase (DCE-MRI); t2 = T2-weighted imaging (T2WI); dwi = diffusion-weighted imaging (DWI, b=800). Feature class abbreviations: GLRLM = Gray Level Run Length Matrix; GLCM = Gray Level Co-occurrence Matrix; NGTDM = Neighborhood Gray-Tone Difference Matrix; GLDM = Gray Level Dependence Matrix. LoG = Laplacian of Gaussian. Wavelet sub-band labels (LHL, LHH, HLL) follow the standard three-letter convention indicating low/high frequency decomposition in each dimension.

**Distribution across MRI sequences:** T2WI: 1 feature; DWI: 1 feature; Arterial phase: 7 features; Portal venous phase: 3 features; Delayed phase: 4 features.
