# Supplementary material for: Predictive value of an AI model integrating MRI radiomics and clinical features for early recurrence of hepatocellular carcinoma prior to radiofrequency ablation
Source: Front Radiol. 2026 Aug 18;6:1824091. doi: 10.3389/fradi.2026.1824091 (PMC13530903; doi:10.3389/fradi.2026.1824091)
Supplement: Supplementary file 2 [file Supplementaryfile2.docx]

# **Supplementary Table S2**

**Confusion Matrix of the RF-based Integrated Model at the Optimal Threshold (0.42) in the Internal Hold-out Test Set**

|  | **Predicted Recurrence** | **Predicted No Recurrence** | **Total** |
| --- | --- | --- | --- |
| **Actual Recurrence** | **TP = 10** | **FN = 2** | **12** |
| **Actual No Recurrence** | **FP = 4** | **TN = 18** | **22** |
| **Total** | **14** | **20** | **34** |

**Performance Metrics at Optimal Threshold (0.42):**

Sensitivity (Recall) = TP / (TP + FN) = 10 / (10 + 2) = 0.833

Specificity = TN / (TN + FP) = 18 / (18 + 4) = 0.818

Accuracy = (TP + TN) / (TP + TN + FP + FN) = (10 + 18) / 34 = 0.824

Precision (PPV) = TP / (TP + FP) = 10 / (10 + 4) = 0.714

NPV = TN / (TN + FN) = 18 / (18 + 2) = 0.900

F1 score = 2 × TP / (2 × TP + FP + FN) = 2 × 10 / (2 × 10 + 4 + 2) = 0.769

Note: The optimal threshold (0.42) was determined by maximizing the Youden index (Sensitivity + Specificity - 1) from the receiver operating characteristic curve of the RF-based integrated model. TP = true positive; FN = false negative; FP = false positive; TN = true negative; PPV = positive predictive value; NPV = negative predictive value; RF = random forest.
